# Supplementary material for: Global warming-induced upper-ocean freshening and the intensification of super typhoons
Source: Nat Commun. 2016 Nov 25;7:13670. doi: 10.1038/ncomms13670 (PMC5133693; doi:10.1038/ncomms13670)
Supplement: Supplementary Information — Supplementary Figures 1-4, Supplementary Tables 1-2 and Supplementary Notes 1-2. [file ncomms13670-s1.pdf]

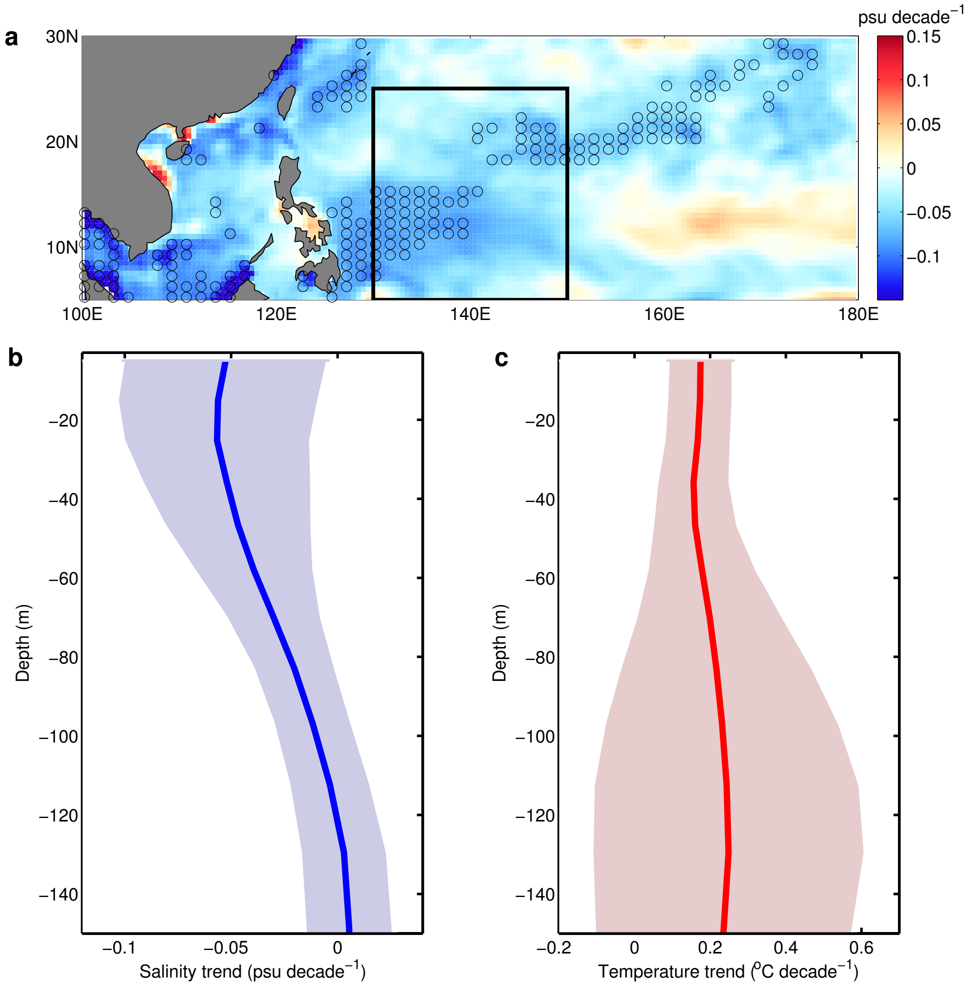

Supplementary Figure 1. Observed trends in upper-ocean salinity and temperature for the 51-year period 1958-2008 based on SODA 2.1.6. (a) Trends in typhoon-season (June-Nov) mean sea surface salinity in psu decade^{-1}. Locations with statistically significant trends are marked with circles. The box in black indicates the study region: 130°E-150°E and 5°N-25°N. Trends in typhoon-season mean subsurface salinity (in psu decade^{-1}) and subsurface temperature (in ^{o}C decade^{-1}), averaged over the region 130°E-150°E and 5°N-25°N (see box in Supplementary Figure 1a). In (b) and (c), the line represents the median trend value and the shaded area indicates the 95% confidence intervals, estimated using a linear regression.

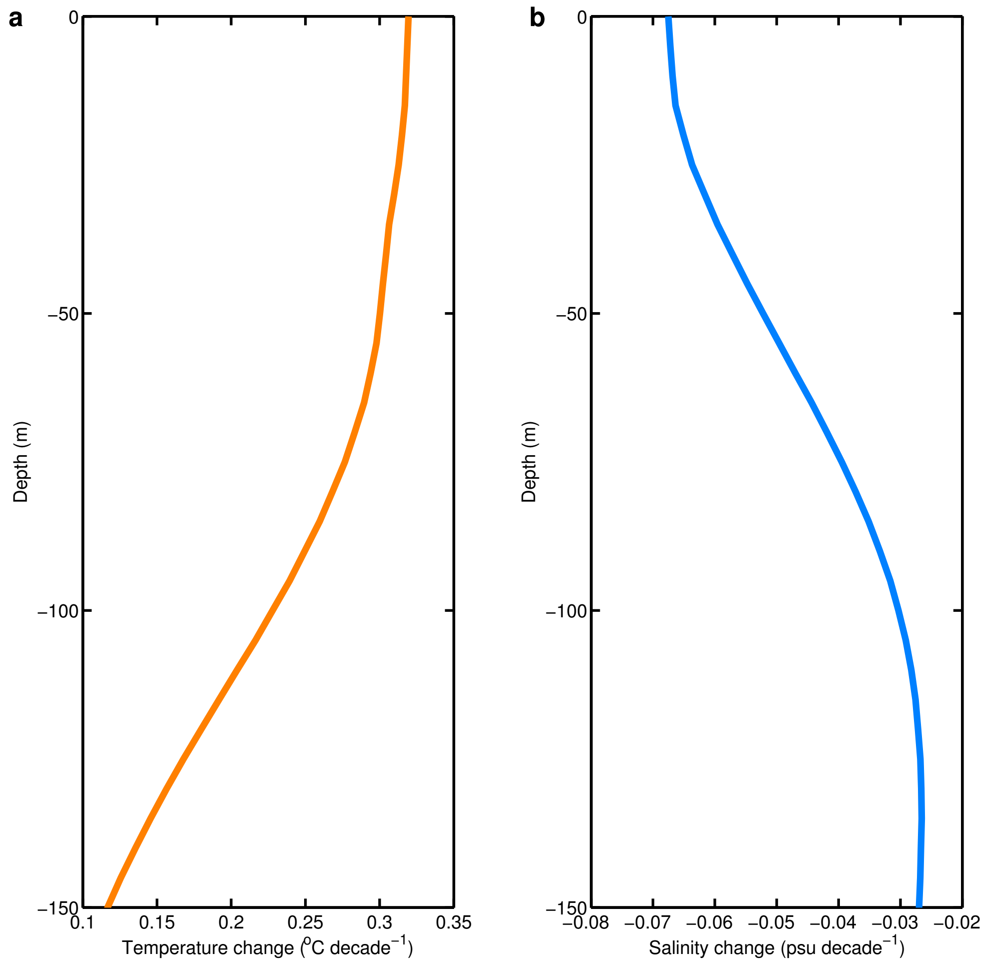

Supplementary Figure 2. Projected 100-year changes in subsurface temperature and salinity based on the CMIP5 multi-model ensemble mean. Changes in (a) subsurface temperature (^{o}C decade^{-1}) in red and (b) subsurface salinity (psu decade^{-1}) in blue, averaged over the region 130°E-150°E and 5°N-25°N (see box in Supplementary Figure 1a), based on the multi-model ensemble mean of eighteen different coupled climate models from CMIP5 under the RCP 8.5 scenario. Change is defined as the difference between the mean over the 20-year periods 2081-2100 and 1981-2000. Changes at all depths are statistically significant at the 95% level.

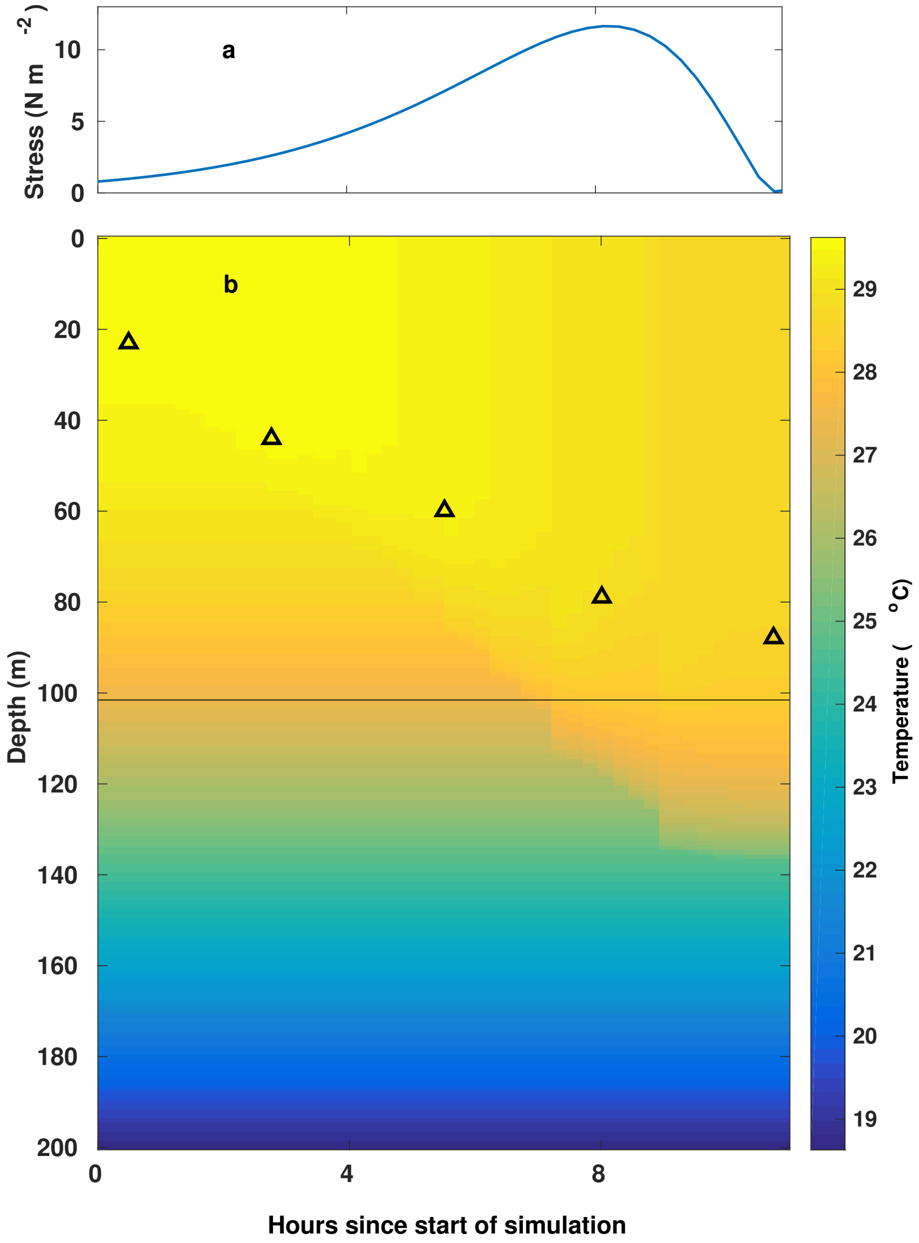

Supplementary Figure 3. Comparison of mixing lengths from PWP and our formula (a) Magnitude of wind stress used to force the PWP model. (b) Evolution of ocean temperature during a typical PWP model run, using initial conditions from September and from the center of the western Pacific region used in the study (see box in Supplementary Figure 1a). Triangles indicate the depth of the mixed layer. The horizontal black line represents the mixing length from our formula.

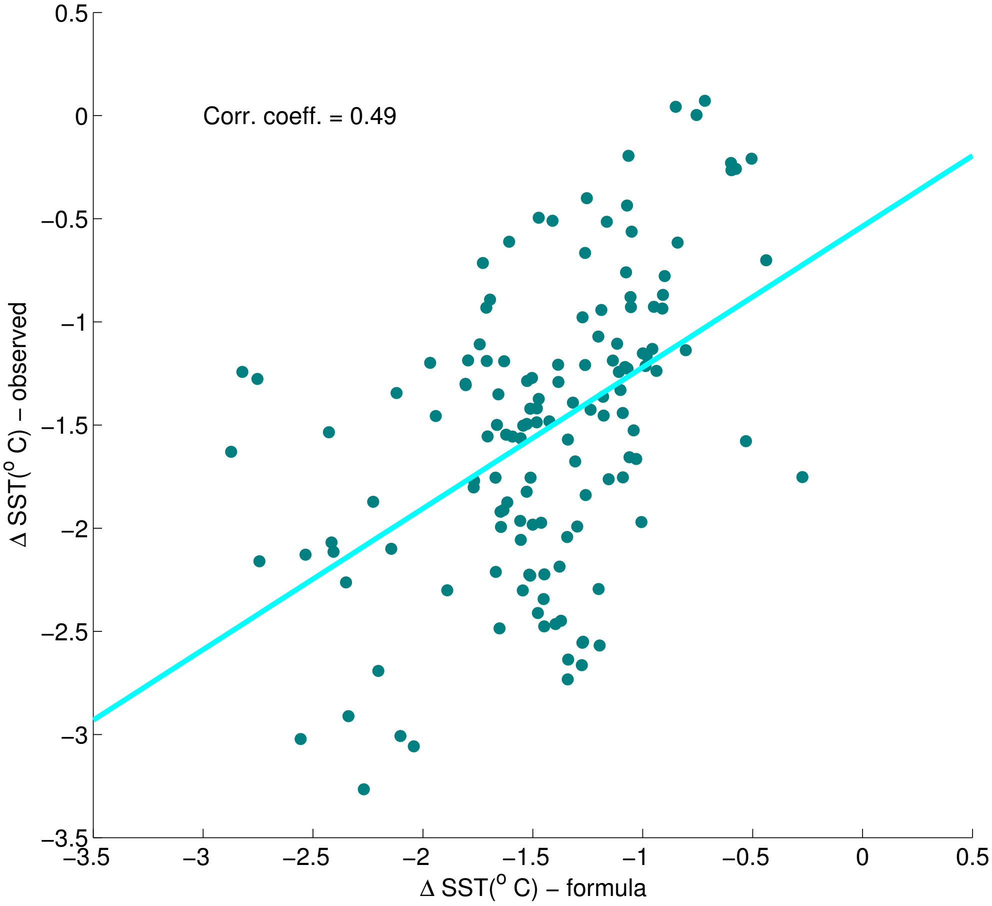

Supplementary Figure 4. Comparison of STY cold wakes estimated using two different methods. Cold wakes estimated using monthly mean oceanic temperature and salinity profiles based on EN4 objective analysis (x-axis) plotted against cold SST wakes estimated using daily microwave satellite SST data (y-axis) for the 11-year period 1998-2008. The regression line is shown in cyan and the correlation coefficient, significant at the 95% level, is indicated.

Supplementary Table 1. Analysis of typhoon track data (1979-2013) Mean SST cooling (Δ SST) in ^{o}C and the mean Dynamic Potential Intensity (DPI) in m s^{-1}, with and without including salinity, obtained using EN4 and ERA-Interim monthly mean data. The differences with and without salinity, for ΔSST and DPI, are statistically significant at the 95% level based on a Student's t-test for difference of means.

**EN4 and ERA-Interim**	**Δ SST**	**Δ SST without salinity**	***DPI***	***DPI* without salinity**
**Super typhoons**	-1.49	-1.90	57.24	52.15
**Tropical storms and typhoons**	-0.74	-0.95	65.45	63.18

Supplementary Table 2. Validation of our formula to accurately represent salinity impacts on super typhoon mixing lengths and cold wakes.

	**With salinity**	**Without salinity**
**PWP mixing length (m)**	76	87.1
**Formula mixing length (m)**	93.5	107.9
**PWP cold wake (^{o}C)**	-1.41	-1.71
**Formula cold wake (^{o}C)**	-1.24	-1.62

Supplementary Note 1. Validation of formula mixing lengths and cold wakes using the PWP model

The numerical experiments with the PWP model show that on average our formula produces a mixing length that is about 23% larger than the final mixed layer depth (the value at the end of each simulation) produced by the PWP model (93.5 m from our formula, 76.0 m from PWP). The reduction in mixing length when salinity is included is likewise similar (14.4 m for our formula versus 11.1 m in PWP), and the percentage reduction is nearly the same (14.6% for PWP, 15.4% for our formula). These similarities occur despite differences in the representation of mixing in our formula, which is based on the Monin-Obukhov length, and the PWP model, which uses the bulk and gradient Richardson numbers. Our formula solves only for the mixing length, the depth to which mixing is confined, and not the full vertical profiles of temperature and salinity as in the PWP model (Supplementary Fig. 3). For this reason, the final PWP mixed layer and our formula's mixing length do not agree perfectly. However, because the Monin-Obukhov mixing length is on the same order as the mixed layer depth, the cold SST wakes are similar when calculated from the PWP model and our formula (-1.41^{o}C for PWP compared to -1.24^{o}C for our formula). The reductions in cold wake magnitude due to salinity are also comparable (0.38^{o}C in our formula and 0.30^{o}C in PWP). An additional factor contributing to the good agreement between the PWP model and our formula is the short-duration, high-intensity, wind-driven mixing associated with a STY, for which the more complicated depth- and time-dependent vertical mixing in the PWP model can be approx-

imated well by the simpler energy balance employed by our formula. These results are summarized in Supplementary Table 2. Thus, there are reasonable agreements between the mixing lengths and SST cold wakes from our model and those from the PWP simulations. Similarly, the sensitivities of the mixing lengths and cold wakes to salinity are comparable. For all parameters, the agreement is within $\pm 27\%$.

Supplementary Note 2. Validation of formula cold wakes using satellite observations

For further validation of our formula's ability to represent cold wakes realistically, we compared our formula's cold SST wakes to the cold wakes measured by satellite microwave sensors, based on the observed tracks of all STYs in our study region (see Figure 1a) during 1998-2008. We used a 4° x 4° region centered on the storm to calculate the difference between the mean satellite SST three days before and one day after the storm's passage. Monthly mean temperature and salinity profiles from EN4 were used for calculation of cold wakes using our formula. We found good agreement between the mean cold wakes: -1.44^{o}C for our formula and -1.52^{o}C for satellite data (Supplementary Fig. 4). They are correlated at nearly 0.5, a value statistically significant at the 99% level.
